# Reasons for poor follow-up of diabetic retinopathy patients after screening in Tanzania: a cross-sectional study

**DOI:** 10.1186/s12886-016-0288-z

**Published:** 2016-07-19

**Authors:** Christina Mtuya, Charles R. Cleland, Heiko Philippin, Kidayi Paulo, Bernard Njau, William U. Makupa, Claudette Hall, Anthony Hall, Paul Courtright, Declare Mushi

**Affiliations:** Kilimanjaro Christian Medical University College, Faculty of Nursing, Moshi, Tanzania; Eye Department, Kilimanjaro Christian Medical Centre, Moshi, Tanzania; International Centre for Eye Health, Faculty of Infectious & Tropical Diseases, London School of Hygiene & Tropical Medicine, London, UK; Department of Work & Social Psychology, University Maastricht, Faculty of Psychology and Neuroscience, P.O. Box 616, 6200 MD Maastricht, The Netherlands; Newcastle Eye Hospital Research Foundation, 182 Christo Road, Waratah, NSW 2289 Australia; Kilimanjaro Centre for Community Ophthalmology, Division of Ophthalmology, University of Cape Town, Cape Town, South Africa

**Keywords:** Diabetic retinopathy, Africa, Screening, Follow-up

## Abstract

**Background:**

Diabetes is an emerging public health problem in sub-Saharan Africa. Diabetic retinopathy is the commonest microvascular complication of diabetes and is a leading cause of blindness, mainly in adults of working age. Follow-up is crucial to the effective management of diabetic retinopathy, however, follow-up rates are often poor in sub-Saharan Africa. The aim of this study was to assess the proportion of patients not presenting for follow-up and the reasons for poor follow-up of diabetic patients after screening for retinopathy in Kilimanjaro Region of Tanzania.

**Methods:**

All diabetic patients referred to a tertiary ophthalmology hospital after screening for retinopathy in 2012 were eligible for inclusion in the study. A randomly selected group of patients from the community-based diabetic retinopathy screening register were identified; among this group, follow-up was assessed. Interviews were conducted within this group to inform on the reasons for poor follow-up.

**Results:**

Among the 203 patients interviewed in the study 50 patients (24.6 %) attended the recommended referral appointment and 153 (75.4 %) did not. Financial reasons were self-reported by 35.3 % of those who did not attend the follow-up appointment as the reason for non-attendance. Multiple logistic regression analysis showed that the patient report of the clarity of the referral process (*p* = 0.014) and the patient report of whether a healthcare worker told the patient that diabetic retinopathy could be treated (*p* = 0.005) were independently associated with attendance at a follow-up appointment. Income per month was not associated with attendance at a follow-up appointment on multivariate analysis.

**Conclusions:**

Financial factors are commonly cited as the reason for non-compliance with follow-up recommendations. However, the reasons for poor compliance are likely to be more complicated. This study highlights the importance of health system factors. Improving the clarity of the referral process and frequent reminders to patients that diabetic retinopathy can be treated are practical strategies that should be incorporated into screening programmes to increase attendance at subsequent follow-up appointments. The results from this study are applicable to other screening programmes as well as those for diabetic retinopathy.

**Electronic supplementary material:**

The online version of this article (doi:10.1186/s12886-016-0288-z) contains supplementary material, which is available to authorized users.

## Background

Sub-Saharan Africa (SSA) faces a growing epidemic of non-communicable diseases [1]. The International Diabetes Federation predicts that the number of adults with diabetes in Africa will double from 12 to 24 million by 2030 [2]. Diabetic retinopathy is the commonest microvascular complication of diabetes and is the leading cause of blindness in adults of working age in industrialized countries [3]. The economic costs to the individual, family and society are significant.

A key component in the effective management of patients with diabetic retinopathy is the provision of, and adherence to follow-up services [4]. In many low-income countries follow-up rates are low due to poor transportation infrastructure, costs to patients, and failure to communicate the benefits of returning [5]. The impact of screening for diabetic retinopathy is clearly limited by the issue of poor follow-up, especially given the chronic nature of diabetes and its complications [6].

The aim of this study was to assess the proportion of patients failing to comply with follow up recommendations to attend a referral eye department following a screening visit and to identify the reasons for poor compliance with follow-up appointments. It was conducted in a community based diabetic retinopathy screening programme in the Kilimanjaro Region of Tanzania. We sought to assess the influence of individual factors, therapy related factors and health system factors on compliance with follow-up services to help inform the development of effective strategies for improving adherence to follow-up services in similar settings in Africa.

## Methods

The study was carried out under the auspices of the Kilimanjaro Diabetic Programme (KDP). The KDP, functioning since November 2010, screens diabetic patients for retinopathy at 18 diabetic clinics throughout the Kilimanjaro Region, which has a population of around 1.6 million people. The KDP visits each diabetic clinic regularly where enrolled patients are screened with a mobile retinal camera. The 18 diabetic clinics are based at government, church and private hospitals across the 7 districts in Kilimanjaro Region.

Retinal photographs are taken using a Topcon retinal camera. The images are stored on a laptop and later reviewed and graded by an ophthalmology resident at the tertiary ophthalmology referral hospital, Kilimanjaro Christian Medical Centre (KCMC). A consultant ophthalmologist grades 10 % of the images for quality control. Following grading of the retinal photographs, patients are contacted and referred to KCMC Eye Department for further management if necessary.

Following attendance at one of the diabetic clinics, patients receive short lectures from doctors and nursing staff on the complications of diabetes and the potential impact on vision. There are also posters displayed within each diabetic clinic, which further describe the complications of diabetes and the importance of regular eye examinations. Patients are given a diary, which includes advice on the appropriate diet for diabetics and acts as a logbook for appointments. Patients also receive leaflets, which contain written text and cartoons informing patients of the complications of diabetes and the importance of regular eye screening. Patients are encouraged to take the leaflets away with them to use as a reference. As well as the above measures, 2 radio broadcasts per week were transmitted locally informing patients of diabetes, its complications and the importance of eye screening. The radio broadcasts were in Kiswahili by a nurse working with the KDP and were broadcast from November 2012.

Following screening, patients are typically either sent a text message or are phoned 2–4 weeks after their screening event and informed that further investigations and possibly treatments are needed. Patients are advised whether they should attend KCMC within 1 month or within 3 months depending on the severity of their retinopathy. Patients are not told what the treatments may involve and are particularly not told that laser treatment might be required. Patients who have normal results or do not need further investigations are informed of this via text message and are advised to attend another screening event after 1 year at their respective diabetic clinic. Patients can request phone calls rather than text messages informing them of the results of screening if they are unable to read text messages.

This study was carried out between April and June 2013. Patients were considered eligible if they had their screening event in 2012 and if they had been referred to KCMC eye department after their screening event. Patients were categorized as non-attenders at follow-up if they had not attended KCMC Hospital when the interviews were conducted.

In 2012, 1106 patients were screened by the KDP for diabetic retinopathy. Of these, 420 had retinopathy requiring further assessment and were recommended to attend a follow-up appointment at KCMC. We randomly selected 294 of these patients for interview through a simple random sampling technique using computer generated numbers. The selected patients were contacted using details stored on the KDP database and were interviewed at their local hospital during subsequent KDP screening events.

Each patient was interviewed in Kiswahili by a native speaker. The structured questionnaire used in the study was pilot tested on 10 patients at KCMC Hospital prior to its use in order to ensure adequate understanding. The questionnaire consisted of a demographic section including questions on occupation, education and income, followed by sections on patient knowledge of diabetic retinopathy and their experience of healthcare. Each interview lasted approximately 30 min. Data on the time taken to travel from the patient’s village to KCMC and the cost of travel was obtained from a senior local government official from each district. The questionnaire is available as an Additional file 1.

### Statistical analysis

Following the interviews, the data was entered into a Microsoft Access (2007) database and analyzed using STATA version 13.0.

Frequency distributions were used to analyze the demographic characteristics of the study population. Logistic regression analysis was performed to assess the effect of the following variables on compliance with follow-up recommendations: age, gender, income, the cost of travel from the patients village to KCMC, the time taken (in minutes) to travel from the patients village to KCMC, patient report of the clarity of the referral process and patient report of being informed of the treatment options, A backward stepwise selection process was used to determine which variables to include in the multivariate analysis with a with a *p* value of 0.2 as the criterion for entry.

## Results

Of the 294 people invited for interview, the response rate was 71.4 %, meaning a total of 210 patients were interviewed. The non-responders were significantly older with a mean age of 67.4 (95 % CI = 64.6 – 70.2) compared with 62.3 (95 % CI = 60.8 – 63.9) in the responders (*p* = 0.0013). There were no significant gender differences between the responders and non-responders. For the 84 people who did not attend the interview the reasons were as follows: not contactable due to incorrect or missing mobile number on KDP database (*n* = 63), patient had moved away from study area (*n* = 4), death (*n* = 3), hospital inpatient during data collection (*n* = 3) and travelling during data collection (*n* = 3).

Of the 210 people interviewed, 7 were excluded from the analysis due to incomplete interview forms. Of the 203 participants included in the analysis 50 (24.6 %) did attend the recommended referral appointment after being screened and 153 (75.4 %) did not. Participants were categorized as non-compliant with the referral recommendation if they had not attended KCMC by the time of interview. As all participants were screened in 2012, and the interviews took place between April and June 2013, this meant a minimum period of 4 months between screening and categorization as a non-attender.

Of the 203 participants with completed interview forms, the mean age was 62.4 years and the number of male participants was 87 (42.9 %). 50.7 % of participants had an income <30 000 Tanzanian Shillings (TSH) per month (approximately $14) and 79.2 % had a primary level education or less. None of the demographic variables were associated with attendance at the referral hospital (Table 1).

**Table 1:** Characteristics of study participants split by attendance at the recommended follow-up appointment, *n* (%)

	Attendance at follow-up appointment		
	Yes (*n* = 50)	No (*n* = 153)	Odds ratio (95 % CI)
Gender			
Male	26 (29.9)	61 (70.1)	1.0 (reference)
Female	24 (20.7)	92 (79.3)	0.61 (0.32 – 1.16)
		*p = 0.132	
Age			
≤50	11 (34.4)	21 (65.6)	
51–60	18 (26.1)	51 (73.9)	
61–-70	9 (17)	44 (83)	
71–80	8 (23.5)	26 (76.5)	
>80	4 (26.7)	11 (73.3)	
Mean Age	60.9	62.9	
		**χ ^2^ = 1.12, *p* = 0.29	
Monthly Income			
<30 000 TSH	20 (19.4)	83 (80.6)	
30 000 – 50 000 TSH	13 (26)	37 (74)	
50 000 – 70 000 TSH	5 (27.8)	13 (72.2)	
>70 000 TSH	12 (37.5)	20 (62.5)	
		**χ ^2^ = 4.06, *p* = 0.04	
Education			
No formal education	3 (15)	17 (85)	0.57 (0.13 – 2.57)
Primary	29 (25.4)	85 (74.6)	1.12 (0.43 – 2.88)
Incomplete primary	11 (28.2)	28 (71.8)	1.29 (0.43 – 3.84)
Secondary or higher	7 (23.3)	23 (76.7)	1.0 (reference)
		*p = 0.138	
Referral process clear			
Yes	43 (33.6)	85 (66.4)	4.2 (1.85 – 9.54)
No	7 (9.3)	68 (90.7)	1.0 (reference)
		*p < 0.001	
Were you told diabetic retinopathy can be treated			
Yes	33 (37.1)	56 (62.9)	3.36 (1.72 – 6.58)
No	17 (14.9)	97 (85.1)	1.0 (reference)
		*p < 0.001	
Mean time taken to travel from patients village to KCMC (minutes)	60	84	
		*p = 0.016	
Mean cost of travel from patients village to KCMC (Tanzanian Shillings)	2426	3342	
		*p = 0.022	

* *p* values calculated using *t* test for means and chi squared for proportions

** *p* values calculated using chi squared for trend test

*KCMC* Kilimanjaro Christian Medical Centre, *TSH* Tanzanian Shilling

A greater distance and a higher cost of travel from the patient’s village to KCMC Hospital were significantly associated with non-attendance at the recommended follow-up appointment (Table 1). The average cost of travel from a patient’s village to KCMC Hospital was 916 TSH (approximately $0.50) more for those who did not attend a follow-up appointment.

Multiple logistic regression analysis revealed that the patient report of the clarity of the referral process and the patient report of being informed that diabetic retinopathy can be treated were independently associated with attendance at the referral appointment. (Table 2). When participants were asked if the referral process was clear and whether they were informed that diabetic retinopathy can be treated, a binary response of yes or no was recorded for both questions.

**Table 2:** Logistic regression analysis for factors associated with compliance with follow-up recommendations

	Attendance at follow up appointment			
	Univariate analysis		Multivariate analysis	
Potential risk factors	OR (95 % CI)	*p* value	OR (95 % CI)	*p* value
Older age	0.98 (0.96 – 1.01)	0.302	0.99 (0.96 – 1.03)	0.74
Female sex	0.61 (0.32 – 1.16)	0.134	0.65 (0.32 – 1.32)	0.24
More education	1.09 (0.73 – 1.61)	0.68	Not included	
Higher income	1.34 (1.02 – 1.77)	0.038	Not included	
Referral process clear	4.2 (1.85 – 9.54)	0.001	2.93 (1.24 – 6.92)	0.014
Told by healthcare worker that DR can be treated	3.36 (1.72 – 6.58)	<0.001	2.80 (1.38 – 5.71)	0.005
Time taken to travel from village to KCMC	0.99 (0.99–0.99)	0.018	0.99 (0.99 – 1.00)	0.08
Cost of travel from village to KCMC	0.99 (0.99–0.99)	0.025	Not included	

A backward stepwise selection process was performed to decide which factors to include in the multivariate model. A *p* value of <0.2 was the criterion for entry into the multivariate model

*DR* diabetic retinopathy, *KCMC* Kilimanjaro Christian Medical Centre

A *p* value of <0.05 was used to indicate significance

The participants who reported the referral process as clear were significantly younger (OR: 0.97; 95 % CI: 0.95–0.99), more educated (OR: 1.61; 95 % CI: 1.15–2.25), with a higher income (OR: 1.46; 95 % CI: 1.14–1.86) and the journey from their village to KCMC required less time (OR: 0.99; 95 % CI: 0.99-0.99). There were no significant differences between the participants who reported being informed that diabetic retinopathy can be treated versus those who did not.

A total of 76 patients reported the referral process as not clear. When they were asked what was not clear about the referral process 40 % said the treatment cost, 36 % stated the reason for referral and 24 % said the outcome of treatment.

### Self reported reasons for non-attendance at the referral appointment

Of the 153 people who did not attend their referral appointment the commonest reason for non-attendance was reported as financial (35.3 %). The remaining reasons for non-attendance at the referral appointment, in decreasing order of frequency, were: fear of surgery (15.6 %), too busy working (14.3 %), distance to the hospital (10.4 %), escort required (9 %), didn’t see the importance (4.6 %), forgot (3.3 %), can’t leave the house alone (2.6 %), no health insurance (2.6 %), needed permission (1.3 %) and other (1.3 %).

## Discussion

Diabetes and diabetic retinopathy represent an emerging public health problem in SSA [7]. This study is the first to our knowledge to assess the rates of follow-up after screening for diabetic retinopathy and to identify reasons for non-compliance with follow-up recommendations [6].

The adherence to follow-up services has been identified by the World Health Organization (WHO) as a key component in the effective management of diabetic retinopathy [4]. Of the patients interviewed in this study, only 25 % attended their recommended follow-up appointment at KCMC. It is essential that this low rate of follow-up be improved in order to effectively manage and prevent blindness from diabetic retinopathy.

Demographic factors, including age, gender and education were not associated with attendance at the follow-up appointment. Financial reasons were most commonly self reported as the reason for non-attendance however, income was not significantly associated with attendance in the multivariate analysis. In addition, of the patients who reported the referral process as not clear, 40 % reported treatment cost when they were asked what was unclear. The discrepancy between self-reported reasons for non-compliance with eye care and income has previously been studied in relation to cataract surgery [8]. The authors noted a distinction between capacity to pay and willingness to pay with “lack of money” serving as a convenient and acceptable explanation to healthcare workers [8]. This study suggests there may be a similar pattern in relation to diabetic retinopathy.

The effect of indirect costs of treatment on compliance with eye care services in sub-Saharan Africa has previously been reported [9–11]. A study in Ethiopia cited indirect costs as the main barrier to accessing eye care services [9]. This study also suggests that the indirect costs of treatment play a role in patient compliance with follow-up, as evidenced by the associated between travel costs to the referral hospital and attendance. Patients requiring hospital services are frequently older and need escorts to hospital. Of the patients who did not attend a follow-up appointment in this study, 9 % cited the main reason for non-compliance was because they needed an escort. Relatives are often required to travel with elderly patients, possibly with low vision, incurring additional costs and loss of earnings, hence compounding the impact of indirect treatment costs. Strategies to improve the integration between community and hospital care, making travelling to hospitals easier and more affordable for patients and relatives, may help to improve patient compliance with follow-up recommendations.

Health system factors were shown to be significantly associated with compliance with follow-up recommendations. The patient reported clarity of the referral process and the patient report of whether they were told diabetic retinopathy can be treated were both independently associated with attendance.

Improving the clarity of the referral process by explaining the treatment costs, the reasons for referral and the likely health benefits to the patient offer pragmatic ways to maximize the impact of screening programmes through increasing follow-up rates. The results are applicable to diabetic retinopathy screening and other screening programmes in sub-Saharan Africa.

The patients who reported that the referral process was not clear were significantly older, less educated, poorer and lived further from KCMC. This provides additional insight into the groups of patients who are likely to be unclear on referral processes. It is important that these patients are recognized as vulnerable and particular care should be taken to ensure they understand the referral process. In order to limit the impact of poor follow-up, offering laser treatment as an outreach service in peripheral hospitals could be considered.

The importance of frequent reminders that diabetic retinopathy can be treated is highlighted by the significant relationship between patients reporting being told that diabetic retinopathy can be treated and attendance at a follow-up appointment. This suggests that patient education and an understanding of the treatment options is influential is compliance with follow-up recommendations.

The second commonest self reported reason for non-compliance with recommended follow-up was fear of surgery. A number of studies in southern India identified fear of surgery and lack of knowledge about glaucoma as reasons for poor compliance with follow-up and treatment within eye care [12–15]. This further highlights the importance of educating patients within screening programmes to ensure understanding of the disease being screened for, the treatment options and the necessity and benefits of early treatment. Understanding the underlying reasons for patient's concerns and fears may require additional in-depth anthropologic investigations.

A limitation of this study is selection bias. As the study was carried out under the auspices of the KDP, all patients included in the study had registered with the KDP and had attended a screening event. There are an unknown number of diabetic patients in Kilimanjaro Region who do not attend diabetic clinics and therefore are not registered with the KDP; it is likely that these patients would also be non-attenders at follow-up. The response rate to the invitations to interview was 71.4 % adding a further element of selection bias. This meant that a number of patients eligible for interview were not included in the study. This low response rate was largely due to incorrect or missing telephone numbers within the KDP database. This, in itself, highlights the challenges involved in providing follow-up care in sub-Saharan Africa. The influence of selection bias means the results are not generalizable to the diabetic population of Kilimanjaro Region as the study sample was biased towards those interacting with diabetic services. The severity of retinopathy and the visual acuity of participants were not recorded in this study. These factors could potentially have influenced patient compliance with follow-up and is an area for further research. Finally, the results are dependent upon responses to interviews carried out by health care providers; patients may have been unwilling to express their true feelings regarding the health care system and providers.

## Conclusions

In conclusion, a number of factors have previously been highlighted as reasons for poor attendance at follow-up appointments in eye health in Africa including costs, accessibility and knowledge of services, trust in treatment outcomes and cultural and social barriers [16]. In settings like Kilimanjaro Region, the reasons for poor compliance are likely to be related to a combination of financial, educational and health system factors. Effort is needed to improve integration between community and hospital care, improve the clarity of the referral process particularly targeting vulnerable patients groups and to continually inform and educate patients on the treatment costs, options and likely benefits.

As the number of adults with diabetes in Africa is expected to double by 2030 [2], it is essential that adequate strategies are in place to identify, treat and follow-up patients with retinopathy. Appropriate referral and reliable follow-up of patients are crucial to this process given the chronic nature of diabetes. It is important that the challenges involved in the management of chronic disease in Africa are identified and research aimed at testing strategies to improve follow-up after screening programmes throughout the region would be helpful.

## Abbreviations

KCMC, kilimanjaro christian medical centre; KDP, kilimanjaro diabetic programme; SSA, Sub-Saharan Africa; TSH, Tanzanian Shilling; WHO, World Health Organization

## Additional file

Additional file 1: Study Questionnaire. The interview was conducted in Kiswahili by a native speaker. The structured questionnaire used was pilot tested on 10 patients at Kilimanjaro Christian Medical Centre (KCMC) prior to its use in order to ensure adequate understanding. The answers were recorded in Kiswahili and then translated into English. (DOCX 84 kb)
