# Supplementary material for: Reasons for poor follow-up of diabetic retinopathy patients after screening in Tanzania: a cross-sectional study
Source: BMC Ophthalmol. 2016 Jul 19;16:115. doi: 10.1186/s12886-016-0288-z (PMC4950081; doi:10.1186/s12886-016-0288-z)
Supplement: Additional file 1: — Study Questionnaire. The interview was conducted in Kiswahili by a native speaker. The structured questionnaire used was pilot tested on 10 patients at Kilimanjaro Christian Medical Centre (KCMC) prior to its use in order to ensure adequate understanding. The answers were recorded in Kiswahili and then translated into English. (DOCX 84 kb) [file 12886_2016_288_MOESM1_ESM.docx]

**Appendix**

**Study Questionnaire**

I would like to ask you a few questions in order to get your views on factors influencing compliance with eye health care referral. This session will take at least 20 to 30 minutes. Your name will not be needed.

**Interviewers Name: ………. Date:……………**

District:________________________ Village:___________________Questionnaire No:_____

**PART A: SOCIO-DEMOGRAPHIC CHARACTERISTICS**

| **SN** | **Question** | **Answer** | | **Code** | | **No** |
| --- | --- | --- | --- | --- | --- | --- |
| 01 | Age | […………………………………] | |  | |  |
| 02 | Gender | 1.Male  2.Female | | 01  02 | | [ ] |
| 03 | Marital status | 1. Married 2. Cohabiting 3. Single 4. Divorced/Separated 5. Widow/Widowed | | 01  02  03  04  05 | | [ ] |
| 04 | Education level | 1. Non 2. Primary 3. Incomplete primary 4. Secondary 5. Advanced diploma 6. University degree | | 01  02  03  04  05 | | [ ] |
| 05 | Have you ever had a previous DR referral? | 1. Yes 2. No | | 01  02 | |  |
| 06 | Occupation | 1. Farmer 2. Business person 3. Animal husbandry 4. Employed 5. Other specify | | 01  02  03  04 | | [ ] |
| 07 | Family history of Diabetic | 1. Yes 2. No   If yes who_________ | | 01  02 | | [ ] |
| 08 | How much is your income per month? | 1. Less Tshs 30,000 2. Tshs 30,000 -49,000 3. Tshs 50,000- 70,000 4. Tshs 71-100,000 5. Tshs 100,000+ | | 01  02  03  04  05 | | [ ] |
|  | **PART A: QUESTIONS ON KNOWLEDGE ON DIABETIC RETINOPATHY** | | | | | |
| 9 | Have you ever heard of DR  If yes, what is DR? | | Yes  No  [………………………………] | 01  02 | | [ ] |
| 10 | What are the causes of DR | | 1. Diabetic complication 2. High blood sugar level 3. Poor blood supply to the retina 4. Sijui | 01  02  03  04 | | [ ] |
| 11 | Do you know any symptoms of DR. | | 1. Yes 2. No | 01  02 | | [ ] |
| 12 | What are the risk factors for DR | | 1. Diabetic disease 2. Poor metabolic control 3. Obesity 4. Hypertension 5. Family history 6. Don’t know | 01  02  03  04  05  06 | | [ ] |
| 13 | Is DR preventable? | | 1. Yes 2. No 3. Don’t know | 01  02  03 | | [ ] |
| 14 | If yes what are the prevention measures that you know? | | 1. Health promotion 2. Medical treatment 3. Health education 4. Screening for DR 5. Others (Specify…) | 01  02  03  04  05 | | [ ] |
| 15 | What are the symptoms of DR | | 1. No signs 2. Eye Pain 3. Blurred central vision 4. Macula edema 5. Don’t know | 01  02  03  04  05 | | [ ] |
| 16 | What are the effect/impact of DR | | 1. Blindness 2. Visual impairment 3. Eye problem 4. Death 5. Others specify | 01  02  03  04  05 | | [ ] |
| 17 | Who is at risk of DR? | | 1. Diabetic patients 2. Obese patient 3. Family history 4. Hypertensive patients 5. Other specify | 01  02  03  04  05 | | [ ] |
| 18 | Do you think DR can be treated? | | 1. Yes 2. No 3. I don’t know | 01  02  03 | | [ ] |
| 19 | If Yes, what are the treatment  If NO why? | | 1. Laser 2. Drugs 3. Surgery 4. No treatment   Other (specify[……………] | 01  02  03 | | [ ] |
| 20 | Have you ever sought treatment other than from the modern sector | | 1. Yes 2. No | 01  02 | |  |
| 21 | If yes, mention please | | 1. Religious prayers 2. Traditional healers 3. Herbal medicine | 01  02  03 | |  |
|  | What are your views regarding the effectiveness of the above (ask one by one)? | | ……………………………..  ……………………………… |  | |  |
| 22 | What is your current health status regarding DR? | | 1. Very bad 2. Not very bad 3. Good 4. Don’t know | 01  02  03  04 | |  |
| **PART C: QUESTIONS ON PERCEIVED QUALITY OF CARE** | | | | | | |
| 23 | Have you ever received any DR care at KCMC? | | 1. Yes 2. No | | 01  02 |  |
| 24 | What is your experience with health care providers  **Please give reason for your choice** | | 1. Hospitality 2. , Language 3. Waiting time 4. Time for consultation, 5. Service was good   [……………………………] | | 01  02  03  04  05 |  |
| 25 | How long did you have to wait to see the doctor? | | 1. 30 minutes 2. One hour 3. More than an hour depending on workload 4. Not very long | | 01  02  03  04 |  |
| 26 | Does the health care provider spend enough time to explain about your problem? | | 1.Yes  2. No  3. Don’t remember | | 01  02  03 |  |
| 27 | If yes, what information provided? | | 1.Causes of diabetic retinopathy  2. Impacts of diabetic retinopathy  3.Advantages of early treatment  4. Importance DR screening | | 01  02  03  04 |  |
| 28 | Did health providers tell you diabetic retinopathy can be treated | | 1.Yes  2. No | | 01  02 |  |
| 29 | Was the referral process clear? | | 1.Yes  2. No | | 01  02 |  |
| 30 | If no, what was not clear? | | 1. Reason for referral 2. Treatment cost 3. Treatment outcome | | 01  02  03 |  |
| 31 | Did you attend the referral appointment? | | 1.Yes  2. No | | 01  02 |  |
|  | **If yes what made you comply?** | | 1. **My eye has problem** 2. **Concerned with my health** 3. **Had insurance(NHI)** 4. **My friend got treated** | | 01  02  03  04 |  |
| 32 | If not attend why? | | 1. Financial problems 2. Forgetting 3. Didn’t see the importance, 4. Distance to hospital 5. Can’t leave my house alone 6. Busy working/difficulty permission from work 7. Needed to obtain permission to attend the hospital. 8. Fear for surgery 9. Needed to find an escort to the hospital 10. No health insurance 11. Other | | 01  02  03  04  05  06  07  08  09  10  11 |  |
| 33 | If attended how was it easy to remember? | | 1. Hospital reminder 2. Referral note 3. Through family member | | 01  02  03 |  |

The interview was conducted in Kiswahili by a native speaker. The structured questionnaire used was pilot tested on 10 patients at Kilimanjaro Christian Medical Centre (KCMC) prior to its use in order to ensure adequate understanding. The answers were recorded in Kiswahili and then translated into English.
